# Observations on Rheumatism

**Published:** 1808-10

**Authors:** 


### *To the Editors of the Medical and Physical Journal.*

Gentlemen,

In a work like unto yours, the intention of which is to communicate new and valuable information to the medical world, I am vexed to see papers occasionally introduced, the writers of which appear to have no other object than that of seeing themselves in print. But they should recollect, that the Readers of your Journal ought, and do expect from every writer in it, something original, or at least interesting, in the sciences to which it is particularly appropriated.

Every man who has determined upon employing his pen on any subject in medicine, should first take pains to inform himself of whatever has previously been written respecting it; in order that he might avoid the imputation of being a Copyist, and of publishing as original, what is only so to himself. If, when he has done this, he feels satisfied that what he has to communicate will afford information to scientific men, your valuable Miscellany then becomes a desirable channel for transmitting it to such a class of Readers.

In the last Number of your Journal, I met with a paper on Rheumatism, by Mr. Carter; and having been a great sufferer from the acute species of that disease, I was in hopes to have found something in it that would have satisfied a curiosity, principally excited by many days and nights of extreme suffering, or a remedy by which to palliate its future attacks. Disappointed however in both, I could not but ask, for what purpose can Mr. Carter have written written these Observations, and sent them forth to a Medical Public? If it was with a view of informing them of the symptoms, causes, or method of cure of this painful, and oftentimes tedious disease, I think he has sadly overrated his own talents, and betrayed a mean opinion of their abilities and information.

No one, who has read Cullen, can read Mr. Carter's description of the symptoms of Rheumatism, without immediately perceiving that they are copied, and frequently almost verbatim, from that learned Professor. With respect to the causes of the disease, I do not see that there is any thing in Mr. Carter's observations, that is not likewise mentioned by Dr. Cullen; both are of opinion, that the most common is cold, generally or partially applied to the surface of the body. Mr. C. however, objects to what Cullen "has written concerning the cause," as to him it "appears to be unsatisfactory," and thus explains the Modus Operandi of heat and cold.

"When the body is acted upon by any great stimulus, the sudden abstraction of it is attended with dangerous consequences. Thus the excitability being acted upon by the stimulus of heat, in a considerable degree produces an accelerated motion in the fluids, and rouses up action in vessels which were before torpid. Now cold, which is an abstraction of stimulus, being suddenly applied, must arrest and detain a quantity of fluids in the vessels. By the application of cold, the action of the heart and arteries is weakened, the humours which are arrested in the extreme small vessels have a tendency to putrefaction, the juices become acrid, and irritate the parts, whence proceed the inflammation, pain, and other symptoms of the Rheumatism."

I shall now transmit what is written by Cullen, and objected to by Mr. Carter, that your readers may judge whether they differ as to the operation of cold as a cause for producing this disease. "In the case of Rheumatism, I suppose that the most common remote cause of it, that is, cold applied, operates especially on the vessels of the joints, from these being less covered by a cellular texture than those of the intermediate parts of the limbs. I suppose further, that the application of cold produces a constriction of the extreme vessels on the surface, and at the same time an increase of tone or phlogistic diathesis in the course of them, from which arises an increased impetus of the blood, and at the same time a resistance to the free passage of it, and consequently inflammation and

 pain.

pain. Further, I suppose that the resistance formed, excites the vis medicatrix to a further increase of the impetus of the blood; and to support this, a cold stage arises, a spasm is formed, and a pyrexia and phlogistic diathesis are produced in the whole system."

I have now only to notice the method of cure proposed by Mr. Carter; and here I am surprized to find, not one remedy suggested that is not mentioned by Cullen, either as to internal medicines or external applications. There being then, nothing the least novel or instructive, as it regards either the symptoms, causes, or method of cure, I trust I may be allowed to ask, why Mr. C. published his Observations on Rheumatism? It is not my intention to censure these observations, as their Author has in Cullen great authority for what he has compiled; but to point out to him the inutility of publishing in your Journal what every one, at all conversant in the practice of medicine, must have known. Cullen's work seems to have been his only text book, for he appears to be unacquainted with subsequent Authors, and a method of cure which, in most cases of acute rheumatism that occur in the metropolis, very much shortens the duration of the disease. But this is a subject on which it is not now my intention to enter. My wish is to discourage that great desire of writing, which prompts men to become Authors, and unwittingly committing themselves, to prevent the insertion of such papers as the one I have noticed, and thus to render your valuable Journal still more so to every one who is, like

Your's, &c.

*Sept.* 14, 1808. A CONSTANT READER.

